# Synthesis and Microbiological Evaluation of New 2- and 2,3-Diphenoxysubstituted Naphthalene-1,4-diones with 5-Oxopyrrolidine Moieties

**DOI:** 10.3390/molecules171214434

**Published:** 2012-12-05

**Authors:** Aušra Voskienė, Birutė Sapijanskaitė, Vytautas Mickevičius, Ilona Jonuškienė, Maryna Stasevych, Olena Komarovska-Porokhnyavets, Rostyslav Musyanovych, Volodymyr Novikov

**Affiliations:** 1 Department of Organic Chemistry, Kaunas University of Technology, Radvilėnų pl. 19, LT-50254 Kaunas, Lithuania; E-Mails: ausra.voskiene@ktu.lt (A.V.); birute.sapijanskaite@ktu.lt (B.S.); vytautas.mickevicius@ktu.lt (V.M.); 2 Department of Technology of Biologically Active Substances, Pharmacy Biotechnology, National University “Lviv Politechnic” Bandera str. 12, 79013, Lviv-13, Ukraine; E-Mails: maryna_s@inbox.ru (M.S.); olkomarovska@gmail.com (O.K.-P.); rostykm71@ukr.net (R.M.); vnovikov@polynet.lviv.ua (V.N.)

**Keywords:** 3-substituted 1-(3-hydroxyphenyl)-5-oxopyrrolidine derivatives, 1,4-naphtho-quinone, antibacterial activity, antifungal activity

## Abstract

New 3-substituted 1-(3-hydroxyphenyl)-5-oxopyrrolidine derivatives containing hydrazone, azole, diazole, oxadiazole fragments, as well as 2-phenoxy- and 2,3-diphenoxy-1,4-naphthoquinone derivatives were synthesized. The structure of all compounds has been confirmed by NMR, IR, mass spectra, and elemental analysis data. Methyl 1-{3-[(3-chloro-1,4-dioxo-1,4-dihydro-2-naphthalenyl)oxy]phenyl}-5-oxo-3-pyrrolidinecarboxylate demonstrated potential antibacterial and antifungal activities as determined by diffusion and serial dilution methods, while *N*'-[(4-bromophenyl)methylidene]-1-{3-[(3-chloro-1,4-dioxo-1,4-dihydro-2-naphthalenyl)oxy]phenyl}-5-oxo-3-pyrrolidinecarbohydrazide and 2-{3-[4-(1,2,3-oxadiazol-5-yl)-2-oxo-1-pyrrolidinyl]phenoxy}-3-{3-[4-(1,3,4-oxadiazol-2-yl)-2-oxo-1-pyrrolidinyl]phenoxy}naphthoquinone showed antifungal activity against *Candida tenuis* and *Aspergillus niger* at low concentrations, as determined by the serial dilution method. The substitution of the methoxy fragment by *N-*containing substituents in monophenoxy substituted naphthoquinones was found to decrease their activity against *Mycobacterium luteum*. Besides, introduction of the second phenoxy substituted fragment increased the antifungal activity against *Candida tenuis* and *Aspergillus niger* at lower concentrations.

## 1. Introduction

Quinones are important compounds widely spread in Nature. Compounds containing 1,4-naphthoquinone moiety exhibit a broad spectrum of biological activities such as cytotoxic [1,2] antiviral [3,4] anti-inflammatory [5,6] antimalarial [7], antibacterial [8,9], antifungal [10] and antiproliferative properties [11]. Naphthoquinones are particularly important in dye chemistry, and recently they have been used for new infrared dyes in optical recording media [12,13,14]. The aim of this research was to synthesize some new 2,3-disubstituted 1,4-naphthoquinone derivatives and to screen their antibacterial and antifungal activity.

The incidence of fungal and bacterial infections has increased dramatically in recent years. The widespread use of antifungal and antibacterial drugs and the development of resistance against them of fungal and bacterial infections has led to serious health hazards. The resistance against wide spectrum antifungal and antibacterial agents has prompted the discovery of new antifungal and antibacterial drugs. The amino and thioether derivatives of 1,4-naphthoquinones have extremely rich biological activities because of their redox potentials [15]. The recent pharmacophore modelling approach and three dimensional quantitative structure-activity (3D-QSAR)/comparative molecular similarity indices analysis (CoMSIA) methods applied to 2,3-disubstituted-1,4-naphthoquinones and heterocyclic 1,4-naphthoquinones in human promyelocytic leukemia HL-60 cell line have explained the pronounced cytotoxic activity of these derivatives [11]. The natural naphthoquinone products alkannin and shikonin and their derivatives are active against Gram-positive bacteria such as *Staphylococcus aureus*, *Enterococcus faecium* and *Bacillus subtilis*. 2,3-Diamino-1,4-naphthoquinone itself was found to act as an antibacterial agent against *Staphylococcus aureus*, with IC_50_ values ranging from 30 to 125 µg/mL [16]. The prevalence of strains of *Staphylococcus aureus *resistant to conventional antibiotics has increased to high levels in some hospitals. The biological activity of several well-known and widely used anthracycline antibiotics such as daunomycin and doxorubicin is thought to be associated to the hydroxyquinone structure. Moreover, equivalent active sites are also present in the tetracycline antibiotics as well as in myxopyronin. The antibacterial effect is also related to naphthoquinones from vegetal origin and isoxazolyl-naphthoquinones. In addition, the fungitoxic effect of 1,4-naphto-quinones and the antiviral activity of some hydroxyquinones have been described [17].

Various 2,3-disubstituted 1,4-naphthoquinone derivatives can be prepared from 2,3-dichloro-1,4-naphthoquinone by its reactions with amino- and hydroxy substituted compounds. In our previous study [18], a series 2- and 2,3-disubstituted [2,4-dioxotetrahydropyrimidin-1(2H)-yl)phenoxy]-naphthalene-1,4-diones were synthesized, and some of them exhibited antimicrobial activity against *Staphylococcus aureus*, *Mycobacterium luteum*, *Candida tenuis *and *Aspergillus niger*.

## 2. Results and Discussion

### 2.1. Synthesis and Structural Peculiarities of New Compounds

**Scheme 1:**
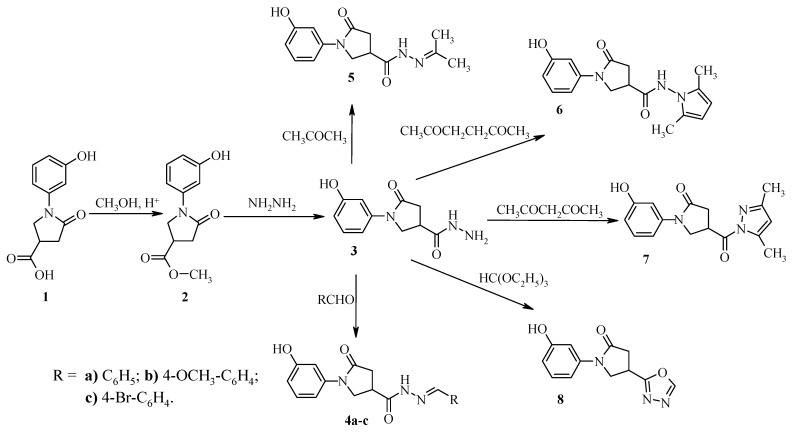
Synthesis of 3-substituted 1-(3-hydroxyphenyl)-5-oxopyrrolidines.

**Scheme 2:**
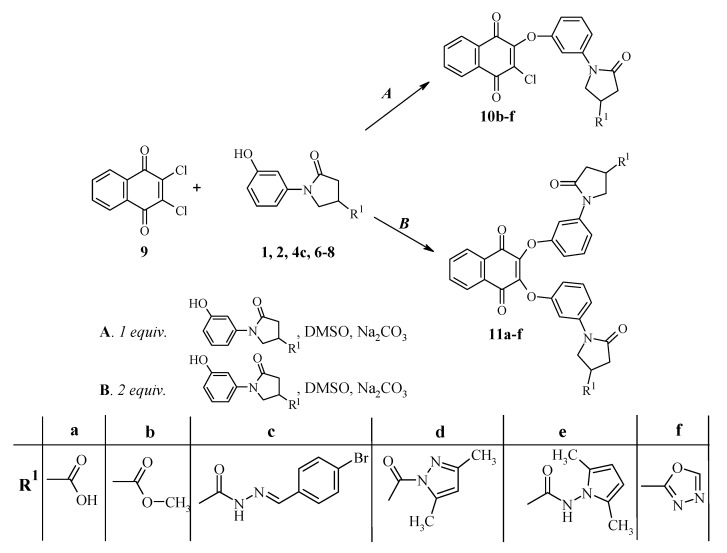
Synthesis of phenoxy-substituted 1,4-naphthoquinone derivatives.

In this work, we describe the synthesis of new 2- and 2,3-diphenoxy-substituted 1,4-naphthquinone derivatives containing hydrazone, azole, diazole and oxadiazole fragments, and investigation their microbiological activity. The target compounds **2**–**11** were synthesized as illustrated in Scheme 1 and Scheme 2. Methyl 1-(3-hydroxyphenyl)-5-oxo-3-pyrrolidinecarboxylate (**2**) was synthesized by esterification of 1-(3-hydroxyphenyl)-5-oxo-3-pyrrolidine carboxylic acid (**1**) with an excess of methanol under reflux in the presence of a catalytic amount of sulphuric acid (Scheme 1). Reaction of ester **2** with hydrazine hydrate in 2-propanol under reflux gave 1-(3-hydroxyphenyl)-5-oxo-3-pyrrolidine-carbohydrazide **3**, which crystallized from the reaction mixture after cooling. Condensation of compound **3** with aromatic aldehydes and acetone gave hydrazone-type derivatives – 1-(3-hydroxyphenyl)-5-oxo-*N*'-(phenylmethylidene)-3-pyrrolidinecarbohydrazides **4a**–**c** and 1-(3-hydroxy- phenyl)-*N*'-(1-methylethylidene)-5-oxo-3-pyrrolidinecarbohydrazide (**5**). Then diketones – 2,4-pentanedione and 2,5-hexanedione – were used in condensation reaction with carbohydrazide **3**, dimethylpyrazole, and dimethylpyrrole derivatives **6**,**7** were thus obtained. The reactions were carried out in 2-propanol in the presence of acetic or hydrochloric acids as catalysts. The formation of heterocyclic systems in compounds **6** and **7** has been confirmed by the characteristic ^1^H-NMR peak signals at 6.23 ppm and 5.65 ppm, attributed to the CH group proton in the dimethylpyrazole moiety and two protons of CH groups in the dimethylpyrrole one, respectively.

Compounds **4a**–**c**, containing amide and azomethine groups, theoretically can exist as an inseparable mixture of four isomers. The amide group determines the splitting of resonances in ^1^H- and ^13^C-NMR spectra due to the restricted rotation around the (*E/Z*) amide bond. The lone pair of the nitrogen atom in the azomethine group affects the neighbouring atoms and causes formation of geometrical isomers (*cis/trans*) [19,20,21]. The ^13^C-NMR spectra of **4a**–**c** exhibited a double set of resonances of CO, N=CH, pyrrolidinone ring carbons and even some of the benzene ring ones. The data presented above allow us to conclude that the *cis/trans* geometrical isomers of the azomethine group are not observed. The *E/Z* ratio of amide conformers can be easily quantified by NMR spectroscopy, and it is about 40/60 for compounds **4a**–**c**. Geometrical isomers are not formed in the case of compound **5**, since two identical terminal methyl substituents are positioned at the double bond; therefore, only a mixture of s-*cis* and s-*trans* rotamers is observed in the ^1^H-NMR spectrum.

The monophenoxy- and diphenoxy substituted naphthoquinone derivatives **10**, **11**, containing different substituents in pyrrolidinone moiety, were synthesized by reactions of 1-(3-hydroxyphenyl)-5-oxopyrrolidine-3-carboxylic acid and their derivatives **1**, **2**, **4c**, **6**–**8** with 2,3-dichloro-1,4-naphtho-quinone (**9**).

3-Substituted 1-[3-(3-chloro-1,4-dioxo-1,4-dihydro-naphthalene-2-yloxy)phenyl]-5-oxopyrrolidine derivatives **10b**–**f** were obtained by stirring the mixture of the respective 1-(3-hydroxyphenyl)-5-oxopyrrolidine-3-carboxylic derivatives **2**, **4c**, **6**–**8**, 2,3-dichloro-1,4-naphthoquinone (**9**), and sodium carbonate in dimethyl sulfoxide for 24 hours at room temperature (Scheme 2). The reaction was finished by diluting the reaction mixture with water, causing the products to precipitate. It was noticed that, along with the target products, small amounts of 2,3-disubstituted derivatives **11** were formed. Therefore, reactions of 2,3-dichloro-1,4-naphthoquinone (**9**) with an excess of hydroxyl-substituted compounds **1**, **2**, **4c**, **6**–**8 **were carried out, affording 1-[3-({3-[3-(4-carboxy-2-oxo-1-pyrrolidinyl)-phenoxy]-1,4-dioxo-1,4-dihydro-2-naphthalenyl}oxy)phenyl]-5-oxo-3-pyrrolidinecarboxylic acid and its derivatives **11a**–**f**.

The structures of all synthesized compounds were confirmed by mass spectrometry, IR, ^1^H-, and ^13^C-NMR spectroscopy data. A comparison of the ^1^H-NMR spectra of **10** and **11** revealed the integral intensities of the proton signals to confirm the formation of monosubstituted naphthoquinone in the first and disubstituted derivative in the second case. These spectra showed identical signals for the functional group protons.

### 2.2. Antimicrobial Evaluation of Synthesized Compounds

The synthesized 2,3-disubstituted naphthalene-1,4-diones **10b**–**f** and **11b**–**f** were evaluated for their antibacterial and antifungal activity against *Escherichia coli В-906*, *Staphylococcus aureus 209-Р*, *Mycobacterium luteum В-917* (as nonpathogenic test bacteria culture representative of the genus *Mycobacterium, *which are classified as acid resistant Gram-positive bacteria, because they have no outer cell membrane in their cell walls), *Candida tenuis VCM Y-70*, and *Aspergillus niger VCM F-1119* strains by the diffusion [22] and serial dilution techniques [23] (determination of minimal inhibition concentrations, MIC). Their activity was compared with that of the known antibacterial agent vancomycin and the antifungal agent nystatin.

As one can see from the data presented in Table 1, *S. aureus* was low-sensitive to monophenoxy-substituted compounds **10b** and **10f** and not sensitive to other mono- and disubstituted naphthoquinones at the investigated concentrations (diffusion method). The bacterial strain *Mycobacterium luteum *was low-sensitive to naphthoquinones **10b** and **11b**, for which the diameter of the inhibition zones at 0.5% concentration was 11.4–12.0 mm. Test-culture *E. coli B-906 *appeared not to be sensitive to the monophenoxy- and diphenoxy-substituted naphthoquinones investigated by the diffusion method. For all compounds **10b**–**f** and **11b**–**f** the growth of bacteria *E. coli* and *S. aureus *was observed at the study concentrations, indicating the absence of biocidic activity of these compounds against the studied bacteria.

**Table 1:** Bactericidal and fungicidal activities of **10b**–**f** and **11b**–**f** determined by diffusion method.

Compound	Concentration, %	Inhibition diameter of microorganism growth, mm			
		*S. aureus*	*M. luteum*	*C. tenuis*	*A. niger*
**10b**	0.5	8.0	11.4	15.0	15.0
	0.1	7.0	8.7	13.0	14.0
**10c**	0.5	0	0	0	7.0
**10f**	0.5	8.0	0	14.4	17.4
	0.1	6.0	0	10.0	14.4
**11b**	0.5	0	12.0	0	6.0
	0.1	0	7.0	0	0
**11c**	0.5	0	0	0	7.0
**11d**	0.5	0	0	0	7.0
**11e**	0.5	0	0	0	7.0
**11f**	0.5	0	0	0	9.0
**C** *	0.1	15.0	18.0	19.0	20.0

* Vancomycin was used as a control in the tests of antibacterial activity of the synthesized compounds and Nystatin was used in the tests of antifungal action.

The evaluation of antifungal activity (diffusion method) has revealed the monophenoxy-substituted naphthoquinones **10b**, **10f** to show a moderate fungicidal effect against *C. tenuis *and *A. niger *at 0.5% concentration (the inhibition zone diameter for strain *C. tenuis *was 15.0 and 14.4 mm and for the one for *A. niger *was 15.0 and 17.4 mm, respectively).

Evaluation by the serial dilution method of compounds **10b**, **10d** and **11f** showed a minimal inhibition action against the *M. luteum *bacterial strain at concentrations of 62*.*5, 250 and 125 μg/mL, respectively (Table 2). Compounds **10b**–**11f** didn’t show antibacterial activity against *E. coli* and *S. aureus *by the serial dilution method.

**Table 2:** Bactericidal and fungicidal activity of **10b**–**f** and **11b**–**f **determined by the serial dilution method.

Compound	MIC, μg/mL		
	*M. luteum*	*C. tenuis*	*A. niger*
**10b**	62.5	62.5	7.8
**10c**	+	1.9	3.9
**10d**	250.0	+	+
**10e**	+	+	+
**10f**	+	31.2	62.5
**11b**	+	31.2	+
**11c**	+	250	+
**11d**	+	62.5	+
**11e**	+	15.6	+
**11f**	125.0	3.9	7.8

+ – growth of microorganisms.

Evaluation by the serial dilution method revealed the MIC for substances **10b**,**f**,**c** to be 1.9–62.5 μg/mL against *C. tenuis*, and for the one for **10b**,**f**,**c** and **11f** to be 3.9–62.5 μg/mL against *A. niger*.

Thus, some promising compounds, especially **10c** and **11f**, with antifungal activity against *Candida tenuis* and *Aspergillus niger* fungi at low concentrations were identified among the synthesized compounds by the serial dilution method. Compounds with antibacterial activity against gram-positive bacteria *M. luteum* at concentrations 62.5–250 μg/mL were identified. The compound **10b** had antibacterial and antifungal activities as determined by diffusion and serial dilution methods.

The substitution of the methoxy fragment by *N*-containing substituents in monophenoxysubstituted naphthoquinones decreased antibacterial activity against *Mycobacterium luteum*. Besides, introduction of the second phenoxy substituted fragment increased the antifungal activity against *Candida tenuis* and *Aspergillus niger* at lower concentrations. Based on these data, the following correlation between structure and antibacterial and antifungal activities of the investigated naphthoquinones was made (diffusion method). For S. aureus:

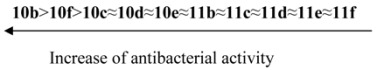

For A. niger:

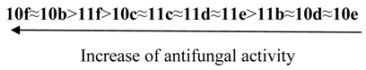



The antimicrobial activity of the tested compounds correlates with their structure. It has been observed that synthesized naphthoquinones containing chlorine atoms in the 2,3-disubstituted naphthoquinone moiety show more significant antibacterial and antifungal activities in comparison with 2,3-diphenoxy-substituted naphthoquinone derivatives. 2,3-Disubstituted derivatives **10b** and **11b** containing methyl carboxylate groups in the pyrrolidinone ring exhibited the highest biological activity. Transformation of this group to hydrazone, dimethylpyrrole, dimethylpyrazole or oxazole substituents decreased the biological activity. 

## 3. Experimental

### 3.1. General

The ^1^H- and ^13^C-NMR spectra were recorded on Varian Unity Inova (300 MHz, 75 MHz) spectrometer operating on Fourier transform mode, using DMSO-*d*_6_ and CDCl_3_ as solvents and TMS as an internal standart (chemical shifts in δ, ppm). IR spectra (ν, cm^−1^) were recorded on a Perkin Elmer Spectrum BX FT–IR spectrometer, KBr tablets. Mass spectra were obtained on Waters ZQ 2000 spectrometer using the atmospheric pressure chemical ionization (APCI) mode and operating at 25 V. Elemental analyses were performed on CE-440 elemental analyzer. Melting points were determined on automatic APA1 melting point apparatus and are uncorrected. TLC was performed on Merck, Silica gel 60 F_254_ (Kieselgel 60 F_254_) silica gel plates.

*Methyl 1-(3-hydroxyphenyl)-5-oxopyrrolidine-3-carboxylate* (**2**). A mixture of 5-oxopyrrolidine **1** (17.7 g, 0.08 mol), methanol (100 mL) and catalytic amount of conc. H_2_SO_4_ was refluxed for 5 h. Then the solvent was evaporated. The precipitated product was neutralized with 5% sodium bicarbonate solution and filtered off, washed with 2-propanol. The crude product was recrystallized from 2-propanol. White crystals, (15.9 g, 85%), m.p. 188–189 °C; ν_max_/cm^−1^ 1660 and 1740 (C=O) and 3159 (OH). ^1^H-NMR (DMSO-*d*_6_): δ_H_ 2.65–2.86 (2H, m, CH_2_CO), 3.38–3.49 (1H, m, CH), 3.67 (3H, s, CH_3_), 3.88–4.06 (2H, m, NCH_2_), 6.52–7.28 (4H, m, H_Ar_), 9.50 (1H, s, OH). ^13^C-NMR (DMSO-*d*_6_) δ_C_ 34.8 (CH), 35.1 (CH_2_CO), 49.8 (CH_2_N), 52.1 (CH_3_), 106.7, 109.8, 111.3, 129.4, 140.0, 157.5 (C_Ar_), 171.4, 173.1 (C=O). MS, *m/z*, (%) = 258 ([M+Na]^+^ 100); Anal. Calcd for C_12_H_13_NO_4_ (235.24): C, 61.27; H, 5.57; N, 5.95. Found: C, 61.34; H, 5.51; N, 5.89.

*1-(3-Hydroxyphenyl)-5-oxopyrrolidine-3-carbohydrazide* (**3**). A mixture of ester **2** (11.0 g, 0.047 mol), 2-propanol (100 mL) and 98% hydrazine hydrate (8.0 g, 0.16 mol) was refluxed for 5 h. The reaction mixture was cooled, the precipitate was filtered off, washed with 2-propanol and purified by recrystallization from water. White crystals, (10.1 g, 92%), m.p. 208–209 °C; ν_max_/cm^−1^ 1697 and 1660 (C=O), 3131 (OH), 3314 and 3253 (NH, NH_2_). ^1^H-NMR (DMSO-*d*_6_): δ_H_ 2.55–2.75 (2H, m, CH_2_CO), 3.07–3.19 (1H, m, CH), 3.74–3.98 (2H, m, NCH_2_), 4.29 (2H, br. s, NH_2_), 6.52–7.26 (4H, m, H_Ar_), 9.27 (1H, s, NH), 9.48 (1H, s, OH). ^13^C-NMR (DMSO-*d*_6_) δ_C_ 33.9 (CH), 35.8 (CH_2_CO), 50.7 (CH_2_N), 106.6, 109.7, 111.2, 129.4, 140.2, 157.5 (C_Ar_), 171.6, 171.9 (C=O). MS, *m/z*, (%) = 258 ([M+Na]^+^ 100); Anal. Calcd for C_11_H_13_N_3_O_3_ (235.24): C, 56.16; H, 5.57; N, 17.86. Found: C, 56.22; H, 5.32; N, 17.84.

### 3.2. General Synthetic Procedure for the Synthesis of Hydrazones ***4a–c***

A mixture of hydrazide **3** (3.5 g, 0.015mol), the corresponding aldehyde (0.016 mol) and 2-propanol (30 mL) was refluxed for 3 h. The reaction mixture was cooled, the precipitate was filtered off and washed with 2-propanol.

*1-(3-Hydroxyphenyl)-5-oxo-N'-(phenylmethylene)pyrrolidine-3-carbohydrazide* (**4a**). White crystals, (4.36 g, 91%), m.p. 194–195 °C; ν_max_/ cm^−1^ 1590 (C=N), 1664 and 1693 (C=O), 3225 (OH) and 3334 (NH). ^1^H-NMR (DMSO-*d*_6_): δ_H_ (*E/Z*, 40/60) 2.68–2.89 (2H, m, CH_2_CO), 3.27–3.38 (1H, m, CH), 3.89–4.14 (2H, m, NCH_2_), 6.52–7.77 (9H, m, H_Ar_), 8.04, 8.22 (1H, 2s, NCH), 9.47 (1H, s, OH), 11.57, 11.63 (1H, 2s, NH). ^13^C-NMR (DMSO-*d*_6_) δ_C_ 32.7, 34.7, 35.0, 32.7 (CH, CH_2_CO), 50.1, 50.5 (CH_2_N), 106.6, 109.8, 111.1, 126.8, 126.9, 128.8, 129.3, 129.8, 130.0, 134.0, 140.1, 140.2, 143.6, 146.9, 157.5, 168.6, (C_Ar_, NCH), 171.7, 171.9, 173.5 (C=O). MS, *m/z*, (%) = 346 ([M+Na]^+^ 100); Anal. Calcd for C_18_H_17_N_3_O_3_ (323.35): C, 66.86; H, 5.30; N, 13.00. Found: C, 66.75; H, 5.24; N, 13.08.

*1-(3-Hydroxyphenyl)-N'-[(4-methoxyphenyl)methylene]-5-oxopyrrolidine-3-carbohydrazide* (**4b**). White crystals, (4.68 g, 89%), m.p. 200–201 °C (from dioxane); ν_max_/cm^−1^ 1600 (C=N), 1609 and 1661, (C=O), 3088 (OH) and 3231 (NH). ^1^H-NMR (DMSO-*d*_6_): δ_H_ (*E/Z*, 40/60) 2.65–2.85 (2H, m, CH_2_CO), 3.24–3.37 (1H, m, CH), 3.85–4.14 (2H, m, NCH_2_), 6.51–7.69 (8H, m, H_Ar_), 7.98, 8.16 (1H, 2s, NCH), 9.47 (1H, s, OH), 11.44, 11.50 (1H, 2s, NH). ^13^C-NMR (DMSO-*d*_6_) δ_C_ 32.7, 34.7, 35.0, 35.8 (CH, CH_2_CO), 50.1, 50.6 (CH_2_N), 55.2 (OCH_3_), 106.6, 109.8, 111.1, 114.3, 126.7, 128.4, 128.6, 129.3, 140.2, 143.4, 146.8, 157.5, 160.6, 160.8, 168.4 (C_Ar_, N=CH), 171.8, 171.9, 173.2 (C=O). MS, *m/z*, (%) = 376,6 ([M+Na]^+^ 100); Anal. Calcd for C_19_H_19_N_3_O_4_ (353.38): C, 64.58; H, 5.42; N, 11.89. Found: C, 64.42; H, 5.39; N, 11.79.

*1-(3-Hydroxyphenyl)-N'-[(4-bromophenyl)methylene]-5-oxopyrrolidine-3-carbohydrazide* (**4c**). White crystals, (5.27 g, 88%), m.p. 255–256 °C (from dioxane); ν_max_/cm^−1^ 1599 (C=N), 1663 and 1698 (C=O), 2973 (OH) and 3172 (NH). ^1^H-NMR (DMSO-*d*_6_): δ_H_ (*E/Z*, 40/60) 2.64–2.89 (2H, m, CH_2_CO), 3.26–3.40 (1H, m, CH), 3.85–4.18 (2H, m, NCH_2_), 6.49–7.70 (8H, m, H_Ar_), 8.01, 8.19 (1H, 2s, NCH), 9.47 (1H, s, OH), 11.63, 11.71 (1H, 2s, NH). ^13^C-NMR (DMSO-*d*_6_) δ_C_ 32.7, 34.7, 34.9, 35.7 (CH, CH_2_CO), 50.0, 50.5 (CH_2_N), 106.6, 109.8, 111.1, 123.0, 123.3, 128.7, 128.9, 129.3, 131.7, 133.4, 140.1, 140.2, 142.4, 145.7, 157.5 (C_Ar_), 171.6, 171.9 173.6 (C=O). MS, *m/z*, (%) = 403 ([M+H]^+^ 95), 405 ([M+H+2]^+^ 100). Anal. Calcd for C_18_H_16_BrN_3_O_3_ (402.25): C, 53.75; H, 4.01; N, 10.45. Found: C, 53.31; H, 4.19; N, 10.71.

*1-(3-Hydroxyphenyl)-N'-isopropylidene-5-oxopyrrolidine-3-carbohydrazide* (**5**). A mixture of compound **3** (1.0 g 4.3 mmol) and dry 2-propanone (30 mL) was refluxed for 4 h. The solvent was evaporated under vacuum, and the precipitated product was filtered off, washed with ethyl ether and purified by recrystallization from 2-propanol. White crystals, (0.8 g, 68%), m.p. 185–186 °C; IR ν_max_/cm^−1^ 1597 (C=N), 1675, 1659 (C=O), 3180 (OH) and 3235 (NH). ^1^H-NMR (DMSO-*d*_6_): δ_H_ (*E/Z*, 55/45)1.87, 1.88 (3H, 2s, CH_3_), 1.93 (3H, s, CH_3_), 2.59–2.80 (2H, m, CH_2_CO), 3.34–3,47 (1H, m, CH), 3.77–4.06 (2H, m, NCH_2_), 6.51–7.29 (4H, m, H_Ar_), 9.46 (1H, s, OH), 10.21, 10.30 (1H, 2s, NH). ^13^C-NMR (DMSO-*d*_6_) δ_C_ 17.1, 17.6, 24.9, 25.2 (2CH_3_), 32.8, 34.2, 35.1, 35.9 (CH, CH_2_CO), 50.2, 50.9 (CH_2_N), 106.6, 106.6, 109.7, 109.8, 111.1, 129.3, 140.2, 140.3, 151.2, 156.1, 157.4, 168.6 (C_Ar_), 171.9, 172.0, 173.6 (C=O). MS, *m/z*, (%) = 298.6 ([M+Na]^+^ 100); Anal. Calcd for C_14_H_17_N_3_O_3_ (275.31): C, 61.08; H, 6.22; N, 15.26. Found: C, 60.98; H, 6.31; N, 15.30.

*N-(2,5-Dimethyl-1H-pyrrol-1-yl)-1-(3-hydroxyphenyl)-5-oxopyrrolidine-3-carboxamide* (**6**). To a solution of hydrazide **3** (3.0 g, 0.013 mol) in 2-propanol (40 mL) 2,5-hexanedione (4.6 g, 0.04 mol) and glacial acetic acid (3 mL) were added. The reaction mixture was stirred and refluxed for 6 h. Then it was cooled to room temperature. The precipitated product was filtered off, washed with ethyl ether and recrystallized from 2-propanol. White crystals, (2.8 g, 70%), m.p. 170–171 °C; ν_max_/cm^−1^ 1603, 1676 (C=O), 3046 (OH) and 3247 (NH). ^1^H-NMR (DMSO-*d*_6_): δ_H_ 1.99 (6H, s, 2CH_3_), 2.67–2.94 (2H, m, COCH_2_), 3.39–3.51 (1H, m, CH), 3.88–4.15 (2H, m, NCH_2_), 5.65 (2H, 2, 2CH), 6.53–7.28 (m, 4H, H_Ar_), 9.51 (1H, s, OH), 10.92 (1H, s, NH). ^13^C-NMR (DMSO-*d*_6_) δ_C_ 10.8 (2CH_3_), 33.9 (CH), 35.7 (CH_2_CO), 50.4 (CH_2_N), 103.0, 106.7, 109.9, 111.3, 126.7, 129.4, 140.1, 157.5 (C_pyrrole_, C_Ar_), 171.4, 171.8 (2C=O). MS, *m/z*, (%) = 336 ([M+Na]^+^ 100); Anal. Calcd for C_17_H_19_N_3_O_3_ (313.36): C, 65.16; H, 6.11; N, 13.41. Found: C, 65.22; H, 6.06; N, 13.45.

*4-[(3,5-Dimethyl-1H-pyrazol-1-yl)carbonyl]-1-(3-hydroxyphenyl)pyrrolidin-2-one* (**7**). A mixture of hydrazide **3** (3.0 g, 0.013 mol), 2,4-pentanedione (4.0 g, 0.04 mol), 2-propanol (30 mL) and a catalytic amount of hydrochloric acid was refluxed for 6 h. The solvent was evaporated under vacuum to dryness, and the oily product was triturated with ethyl ether. The obtained solid was filtered off and washed with ethyl ether. The crude product was purified by recrystallization from 2-propanol. White crystals, (2.0 g, 53%), m.p. 165–166 °C; ν_max_/cm^−1^ 1601 (C=N), 1663, 1722, (C=O) and 3148 (OH). ^1^H-NMR (DMSO-*d*_6_): δ_H_ 2.21, (3H, s, CH_3_), 2.48 (3H, s, CH_3_), 2.76–2.94 (2H, m, COCH_2_), 3.94–4.20 (2H, m, NCH_2_), 4.39–4.51 (1H, m, CH), 6.23(1H, s, CH), 6.52–7.25 (4H, m, H_Ar_), 9.49 (1H, s, OH). ^13^C-NMR (DMSO-*d*_6_) δ_C_ 13.5, 13.9 (2CH_3_), 35.2 (CH), 35.2 (CH_2_CO), 50.1 (CH_2_N), 106.7, 109.9, 111.2, 111.5, 129.3, 139.9, 143.8, 152.0, 157.4 (C_pyrazole_, C_Ar_), 171.4, 171.5 (C=O). MS, *m/z*, (%) = 322 ([M+Na]^+^ 100); Anal. Calcd for C_16_H_17_N_3_O_3_ (299.34): C, 64.20; H, 5.72; N, 14.04. Found: C, 64.31; H, 5.75; N, 14.08.

*1-(3-Hydroxyphenyl)-4-[1,3,4]oxadiazol-2-yl-pyrrolidin-2-one* (**8**). A mixture of compound **3** (7.0 g, 0.03 mol), triethyl orthoformate (35.6 g, 0.24 mol) and *p*-toluenesulfonic acid (1.14 g, 6 mmol) was refluxed for 8 h. After cooling, the precipitate was filtered off, washed with ethyl ether and purified by recrystallization from 2-propanol. White crystals, (3.32 g, 46%), m.p. 181–182 °C; ν_max_/cm^−1^ 1600 (C=N), 1679 (C=O) and 3184 (OH). ^1^H-NMR (DMSO-*d*_6_): δ_H_ 2.81–3.12 (2H, m, CH_2_CO), 4.02–4.31 (3H, m, NCH2, CH), 6.52–7.27 (4H, m, H_Ar_), 9.22 (1H, s, N=CH), 9.48 (1H, s, OH); ^13^C-NMR (DMSO-*d*_6_) δ_C_ 27.5 (CH), 35.9 (CH_2_CO), 50.6 (CH_2_N), 106.9, 109.9, 111.4, 129.3, 139.8, 154.7, 157.4, 166.4 (C_oxadiazole_, C_Ar_), 170.9 (C=O); MS, *m/z*, (%) = 268 ([M+Na]^+^ 100); Anal. Calcd for C_12_H_11_N_3_O_3_ (245.24): C, 58.77; H, 4.52; N, 17.13. Found: C, 58.59; H, 4.41; N, 17.19.

### 3.3. General Synthetic Procedure for the Synthesis of Compounds ***10b–f***

A mixture of 2,3-dichloro-1,4-naphthoquinone **9** (0.88 g 3.9 mmol), the corresponding 1-(3-hydroxyphenyl)-5-oxopyrrolidine derivative **2**, **4c**, ** 6**–**8** (3.9 mmol), sodium carbonate (1.0 g) in dimethyl sulfoxide (15 mL) was stirred at room temperature for 24 h. The reaction mixture was diluted with water (70 mL), the precipitate was filtered off and washed with water.

*Methyl 1-{3-[(3-chloro-1,4-dioxo-1,4-dihydro-2-naphthalenyl)oxy]phenyl}-5-oxo-3-pyrrolidinecarboxylate* (**10b**). The crude product was purified by chromatography on a silica gel 60 column (2-propanone-hexane, 1:1), *R_f_* 0.56. Yellow crystals, (1.0 g, 61%), m.p. 144–145 °C; ν_max_/cm^−1^ 1663, 1679, 1692 and 1736 (C=O). ^1^H-NMR (DMSO-*d*_6_): δ_H_ 2.66–2.86 (2H, m, COCH_2_), 3.38–3.50 (1H, m, CH), 3.66 (3H, s, CH_3_), 3.91–4.11 (2H, m, NCH_2_), 6.92–8.15 (8H, m, H_Ar_). ^13^C-NMR (DMSO-*d*_6_) δ_C_ 34.8 (CH), 35.1 (CH_2_CO), 49.7 (CH_2_N), 52.1 (OCH_3_), 107.3, 111.6, 114.3, 126.5, 126.6, 129.7, 130.5, 131.3, 133.5, 134.4, 134.6, 140.3, 152.3, 156.3 (C_Ar_), 171.8, 172.9, 177.7, 178.1 (C=O). MS, *m/z*, (%) = 449 ([M+Na]^+^ 100), 451 ([M+Na+2]^+^ 35); Anal. Calcd for C_22_H_16_ClNO_6_ (425.83): C, 62.05; H, 3.79; N, 3.29. Found: C, 62.10; H, 3.88; N, 3.18.

*N'-[(4-Bromophenyl)methylidene]-1-{3-[(3-chloro-1,4-dioxo-1,4-dihydro-2-naphthalenyl)oxy]phenyl}-5-oxo-3-pyrrolidinecarbohydrazide* (**10c**). The crude product was purified by chromatography on a silica gel 60 column (ethyl acetate–hexane, 1:1), *R_f_* 0.63. Yellow crystals, (2.1 g, 90%), m.p. 185–186 °C; ν_max_/cm^−1^ 1594 (C=N), 1630, 1676, 1690, 1725 (C=O) and 3215 (NH). ^1^H-NMR (DMSO-*d*_6_): δ_H_ (*E/Z*, 40/60) 2.68–2.91 (2H, m, COCH_2_), 3.30–3.39 (1H, m, CH), 3.93–4.17 (2H, m, NCH_2_), 6.93–8.20 (13H, m, N=CH, H_Ar_), 11.65, 11.71 (1H, 2s, NH). ^13^C-NMR (DMSO-*d*_6_) δ_C_ 32.7, 34.7, 35.0, 35.7 (CH, CH_2_CO), 49.9, 50.4 (CH_2_N), 107.2, 107.4, 111.5, 114.2, 114.3, 123.0, 123.3, 126.4, 126,.6, 128.7, 128.8, 129.7, 130.5, 131.2, 131.7, 133.3, 133.4, 134.4, 134.5, 140.4, 142.4, 145.8, 152.3, 156.3, 168.6 (C_Ar_), 172.1, 172.3, 173.4, 177.7, 178.0 (C=O). Anal. Calcd for C_28_H_19_BrClN_3_O_5_ (592.82): C, 56.73; H, 3.23; N, 7.09. Found: C, 56.85; H, 3.36; N, 6.98.

*2-Chloro-3-{3-[4-(3,5-dimethylpyrazole-1-carbonyl)-2-oxopyrrolidin-1-yl]phenoxy}-[1,4]naphthoquinone* (**10d**). The crude product was recrystallized from ethanol. Yellow crystals, (1.4 g, 72%), m.p. 130–131 °C; ν_max_/cm^−1^ 1572 (C=N), 1667, 1673, 1698 and 1735 (C=O). ^1^H-NMR (DMSO-*d*_6_): δ_H_ 2.19 (3H, s, CH_3_), 2.47 (3H, s, CH_3_), 2.77–2.96 (2H, m, COCH_2_), 3.97–4.24 (2H, m, NCH_2_), 4.40–4.52 (1H, m, CH), 6.21, 6.22 (1H, 2s, C=CH), 6.94–8.15 (8H, m, H_Ar_). ^13^C-NMR (DMSO-*d*_6_) δ_C_ 13.5, 14.0 (2CH_3_), 35.2 (CH), 35.5 (CH_2_CO), 50.2 (CH_2_N), 107.4, 111.6, 114.4, 126.5, 126.6, 129.7, 130.5, 131.3, 133.4, 134.4, 134.6, 140.3, 143.8, 152.1, 152.3, 156.3 (C_pyrazole_, C_Ar_), 171.8, 172.5, 177.7, 178.1 (C=O). MS, *m/z*, (%) = 513 ([M+Na]^+^ 100); 515 ([M+Na+2]^+^ 35) Anal. Calcd for C_26_H_20_ClN_3_O_5_ (489.92): C, 63.74; H, 4.11; N, 8.58. Found: C, 63.65; H, 3.98; N, 8.49.

*1-{3-[(3-Chloro-1,4-dioxo-1,4-dihydro-2-naphthalenyl)oxy]phenyl}-N-(2,5-dimethyl-1H-pyrrol-1-yl)-5-oxo-3-pyrrolidinecarboxamide* (**10e**). The crude product was recrystallized from ethanol. Yellow crystals, (1.8 g, 90%), m.p. 181–182 °C; ν_max_/cm^−1^ 1578 (C=N), 1650, 1676, 1696, 1710 (C=O) and 3313 (NH). ^1^H-NMR (DMSO-*d*_6_): δ_H_ 1.97 (3H, s, CH_3_), 1.99 (3H, s, CH_3_), 2.68–2.96 (2H, m, COCH_2_), 3.40–3.52 (1H, m, CH), 3.94–4.17 (2H, m, NCH_2_), 5.64 (2H, s, 2CH), 6.94–8.16 (8H, m, H_Ar_), 10.88 (1H, s, NH); ^13^C-NMR (DMSO-*d*_6_) δ_C_ 10.8 (2CH_3_), 33.8 (CH), 35.7 (CH_2_CO), 50.2 (CH_2_N), 102.9, 107.4, 111.6, 114.2, 126.4, 126.6, 129.7, 130.5, 131.2, 133.3, 134.4, 134.5, 140.3, 152.3, 156.2 (C_pyrrole_, C_Ar_), 171.6, 171.7, 177.6, 177.9 (C=O). MS, *m/z*, (%) = 527 ([M+Na]^+^ 100); 529 ([M+Na+2]^+^ 35)Anal. Calcd for C_27_H_22_ClN_3_O_5_ (503.95): C, 64.35; H, 4.40; N, 8.34. Found: C, 64.22; H, 4.39; N, 8.40.

*2-Chloro-3-[3-(4-[1,3,4]oxadiazol-2-yl-2-oxopyrrolidin-1-yl)phenoxy]-[1,4]naphthoquinone* (**10f**). The crude product was purified by chromatography on a silica gel 60 column (2-propanone-hexane, 1:1), *R_f_* 0.29. Yellow crystals, (1.4 g, 82%), m.p. 195–196 °C; ν_max_/cm^−1^ 1576, 1597 (C=N), 1678, 1698 and 1743 (C=O). ^1^H-NMR (DMSO-*d*_6_): δ_H_ 2.84–3.12 (2H, m, COCH_2_), 4.06–4.34 (3H, m, CH, NCH_2_), 6.96–8.15 (8H, m, H_Ar_), 9.23 (1H, s, N=CH). ^13^C-NMR (DMSO-*d*_6_) δ_C_ 27.5 (CH), 36.0 (CH_2_CO), 50.6 (CH_2_N), 107.4, 111.7, 114.5, 126.5, 126.6, 129.8, 130.5, 131.3, 133.5, 134.4, 134.6, 140.2, 152.3, 154.9, 156.3, 166.3 (C_Ar_, C_oxadiazole_), 171.4, 177.7, 178.1 (C=O). MS, *m/z*, (%) = 459 ([M+Na]^+^ 100); 461 ([M+Na+2]^+^ 35) Anal. Calcd for C_22_H_14_ClN_3_O_5_ (435.83): C, 60.63; H, 3.24; N, 9.64. Found: C, 60.51; H, 3.31; N, 9.69.

### 3.4. General Synthetic Procedure for the Synthesis of Compounds ***11a–f***

A mixture of 2,3-dichloro-1,4-naphthoquinone **9** (3.3 mmol, 0.75 g), the corresponding 1-(3-hydroxyphenyl)-5-oxopyrrolidine derivative **1**, **2**, **5**–**7**, **8c** (6.6 mmol), sodium carbonate (2.0 g) and dimethyl sulfoxide (15 mL) was stirred at room temperature for 24 h. The reaction mixture was diluted with water (70 mL), the precipitate was filtered off and washed with water.

*1-[3-({3-[3-(4-Carboxy-2-oxo-1-pyrrolidinyl)phenoxy]-1,4-dioxo-1,4-dihydro-2-naphthalenyl}oxy)phenyl]-5-oxo-3-pyrrolidinecarboxylic acid* (**11a**). The crude product was purified by dissolving them in sodium hydroxide solution (5%), filtering the solution, and acidifying the filtrate with acetic acid up to pH 6. Orange crystals, (1.4 g, 72%), m.p. 170–171 °C; ν_max_/cm^−1^ 1671, 1700, 1730, (C=O) and 3072 (OH). ^1^H-NMR (DMSO-*d*_6_): δ_H_ 2.58–2.76 (4H, m, 2COCH_2_), 3.10–3.23 (2H, m, 2CH), 3.80–3.97 (4H, m, 2NCH_2_), 6.84–8.07 (12H, m, H_Ar_). ^13^C-NMR (DMSO-*d*_6_) δ_C_ 35.7 (CH), 35.9 (CH_2_CO), 50.7 (CH_2_N), 107.1, 107.3, 111.7, 113.9, 126.0, 129.3, 130.8, 134.3, 140.2, 145.3, 145.4, 156.4, 156.4 (C_Ar_), 172.6, 174.8, 179.9 (C=O). MS, *m/z*, (%) = 619 ([M+Na]^+^ 100); Anal. Calcd for C_32_H_24_N_2_O_10_ (596.56): C, 64.43; H, 4.06; N, 4.70. Found: C, 64.39; H, 3.98; N, 4.66.

*Methyl 1-{3-[(3-{3-[4-(methoxycarbonyl)-2-oxo-1-pyrrolidinyl]phenoxy}-1,4-dioxo-1,4-dihydro-2-naphthalenyl)oxy]phenyl}-5-oxo-3-pyrrolidinecarboxylate* (**11b**). The crude product was chromatographed over a silica gel 60 column (2-propanone-hexane, 1:1), *R_f_* 0.29. Orange crystals, (0.9 g, 45%), m.p. 90–91 °C; ν_max_/cm^−1^ 1676, 1703 and 1737 (C=O). ^1^H-NMR (DMSO-*d*_6_): δ_H_ 2.64–2.83 (4H, m, 2COCH_2_), 3.35–3.48 (2H, m, 2CH), 3.66 (6H, s, 2CH_3_), 3.84–4.04 (4H, m, 2NCH_2_), 6.88–8.07 (12H, m, H_Ar_). ^13^C-NMR (DMSO-*d*_6_) δ_C_ 34.7 (2CH), 34.9 (2CH_2_CO), 49.7 (2CH_2_N), 52.1 (2OCH_3_), 107.3, 111.8, 113.9, 126.0, 129.4, 130.8, 134.3, 139.9, 145.6, 156.6 (C_Ar_), 171.6, 172.9, 179.9 (C=O). MS, *m/z*, (%) = 647 ([M+Na]^+^ 100); Anal. Calcd for C_34_H_28_N_2_O_10_ (624.61): C, 65.38; H, 4.52; N, 4.48. Found: C, 65.44; H, 4.49; N, 4.52.

*N'-[(4-Bromophenyl)methylidene]-1-{3-[(3-{3-[4-({2-[(4-bromophenyl)methylidene]hydrazino}carbonyl)-2-oxo-1-pyrrolidinyl]phenoxy}-1,4-dioxo-1,4-dihydro-2-naphthalenyl)oxy]phenyl}-5-oxo-3-pyrrolid-inecarbohydrazide* (**11c**). The crude product was chromatographed over a silica gel 60 column (ethyl acetate–methanol, 11:1), *R_f_* 0.58. Yellow crystals, (1.2 g, 38%), m.p. 189–190 °C; ν_max_/cm^−1^ 1591 (C=N), 1678, 1693, 1731 (CO) and 3209 (NH). ^1^H-NMR (DMSO-*d*_6_): δ_H_ (*E/Z*, 40/60) 2.69–2.83 (4H, m, 2COCH_2_), 3.23–3.39 (2H, m, 2CH), 3.84–4.11 (4H, m, 2NCH_2_), 6.86–8.19 (22H, m, 2N=CH, H_Ar_), 11.63, 11.69 (2H, 2s, 2NH). ^13^C-NMR (DMSO-*d*_6_) δ_C_ 32.6, 34.6, 35.0, 35.7, (2CH, 2CH_2_CO), 49.9, 50.4 (2CH_2_N), 107.3, 111.6, 114.0, 123.0, 123.2, 126.0, 128.7, 128.8, 129.3, 130.8, 131.7, 133.3, 134.3, 140.1, 142.4, 145.7, 156.6 (2N=CH, C_Ar_), 172.1, 173.4, 179.9 (C=O). Anal. Calcd for C_46_H_34_Br_2_N_6_O_8_ (958.63): C, 57.64; H, 3.58; N, 8.77. Found: C, 57.51; H, 3.39; N, 8.69.

*2,3-Bis(3-{4-[(3,5-dimethyl-1H-pyrazol-1-yl)carbonyl]-2-oxo-1-pyrrolidinyl}phenoxy)naphthoquinone* (**11d**). The crude product was chromatographed over a silica gel 60 column (2-propanone-hexane, 1:1), *R_f_* 0.72. Yellow crystals, (1.1 g, 43%), m.p. 136–137 °C; ν_max_/cm^−1^ 1587 (C=N), 1677, 1706 and 1723 (C=O). ^1^H-NMR (DMSO-*d*_6_): δ_H_ 2.19, (6H, s, 2CH_3_), 2.46 (6H, s, 2CH_3_), 2.75–2.92 (4H, m, 2COCH_2_), 3.89–4.16 (4H, m, 2NCH_2_), 4.37–4.49 (2H, m, 2CH), 6.22 (2H, s, 2C=CH), 6.88–8.05 (12H, m, H_Ar_). ^13^C-NMR (DMSO-*d*_6_) δ_C_ 13.2, 13.7 (CH_3_), 34.8 (CH), 34.9 (CH_2_CO), 49.8 (CH_2_N), 107.2, 111.3, 111.5, 113.9, 125.8, 129.1, 130.5, 134.1, 139.7, 143.6, 145.3, 151.8, 156.3, (C_pyrrazole_, C_Ar_), 171.4, 172.2, 179.7 (C=O). MS, *m/z*, (%) = 775 ([M+Na]^+^ 100); Anal. Calcd for C_42_H_36_N_6_O_8_ (752.79): C, 67.01; H, 4.82; N, 11.16. Found: C, 67.19; H, 4.91; N, 11.20.

*N-(2,5-Dimethyl-1H-pyrrol-1-yl)-1-[3-({3-[3-(4-{[(2,5-dimethyl-1H-pyrrol-1-yl)amino]carbonyl}-2-oxo-1-pyrrolidinyl)phenoxy]-1,4-dioxo-1,4-dihydro-2-naphthalenyl}oxy)phenyl]-5-oxo-3-pyrrolid-inecarboxamide* (**11e**). The crude product was chromatographed over a silica gel 60 column (2-propanone-hexane, 1:1), *R_f_* 0.33. Orange crystals, (0.6 g, 23%), m.p. 193–194 °C; ν_max_/cm^−1^ 1649, 1676, 1693, 1715 (C=O) and 3252 (NH). ^1^H-NMR (DMSO-*d*_6_): δ_H_ 1.97 (6H, s, 2CH_3_), 1.98 (6H, s, 2CH_3_), 2.65–2.93 (4H, m, 2COCH_2_), 3.37–3.49 (2H, m, 2CH), 3.87–4.11 (4H, m, 2NCH_2_), 5.64 (4H, s, 4CH), 6.90–8.07 (12H, m, H_Ar_), 10.90 (2H, s, 2NH). ^13^C-NMR (DMSO-*d*_6_) δ_C_ 10.6 (4CH_3_), 33.6 (2CH), 35.5 (2CH_2_CO), 49.9 (2CH_2_N), 102.7, 107.1, 111.4, 113.7, 125.8, 126.4, 129.2, 130.5, 134.1, 139.8, 145.4, 156.4 (C_pyrrole_, C_Ar_), 171.4, 179.6 (C=O). MS, *m/z*, (%) = 803 ([M+Na]^+^ 100); Anal. Calcd for C_44_H_40_N_6_O_8_ (780.84): C, 67.68; H, 5.16; N, 10.76. Found: C, 67.59; H, 5.21; N, 10.79.

*2-{3-[4-(1,2,3-Oxadiazol-5-yl)-2-oxo-1-pyrrolidinyl]phenoxy}-3-{3-[4-(1,3,4-oxadiazol-2-yl)-2-oxo-1-pyrrolidinyl]phenoxy}naphthoquinone* (**11f**). The crude product was chromatographed over a silica gel 60 column (2-propanone-hexane, 5:1), *R_f_* 0.23. Orange crystals, (0.52 g, 49%), m.p. 150–151 °C; ν_max_/cm^−1^ 1578, 1604 (C=N), 1677 and 1705 (C=O). ^1^H-NMR (DMSO-*d*_6_): δ_H_ 2.82–3.08 (4H, m, 2COCH_2_), 3.99–4.26 (6H, m, CH, NCH_2_), 6.90–8.07 (12H, m, H_Ar_), 9.21 (2H, s, 2N=CH). ^13^C-NMR (DMSO-*d*_6_) δ_C_ 27.4 (2CH), 35.9 (2CH_2_CO), 50.5 (2CH_2_N), 107.4, 111.9, 114.2, 126.0, 129.4, 130.8, 134.3, 139.9, 145.6, 154.8, 156.6, 166.2 (C_Ar_, C_oxadiazole_), 171.2, 179.9 (C=O). MS, *m/z*, (%) = 667 ([M+Na]^+^ 100); Anal. Calcd for C_34_H_24_N_6_O_8_ (644.41): C, 63.35; H, 3.75; N, 13.04. Found: C, 63.22; H, 3.80; N, 12.97.

### 3.5. Antimicrobial Activity

The synthesized compounds were tested for their *in vitro* antimicrobial and antif*ungal activity* *against* *bacteria Escherichia coli B-906*, *Staphylococcus aureus 209-P*, Mycobacterium luteum B-917 and fungi *Candida tenuis* VCM Y-70, *Aspergillus niger* VCM F-1119 by the diffusion method in agar (method A) and by the serial dilution method (method B).

**Method А**. Determination of antimicrobial and antifungal activity by diffusion method in agar. Antimicrobial and antifungal activity has been tested by diffusion in agar on solid nutrient medium (beef-extract agar for bacteria, wort agar for fungi). Petri plates containing 20 mL of nutrient medium were used for all tested microorganisms. The inoculums (the microbial loading − 10^9^ cells (spores)/1 mL) was spread on the surface of the solidified media and Whatman no.1 filter paper discs (6 mm in diameter) impregnated with the test compound solution (0.1% and 0.5%) were placed on the plates. The duration of bacteria incubation was 24 h at 35 °C and the one of fungi incubation was 48–72 h at 28–30 °C. The antimicrobial effect and degree of activity of the tested compounds were evaluated by measuring the zone diameters. The results were compared with well known drugs (Table 1). Every experiment was repeated three times.

**Method B**. Determination of minimal inhibitory (MIC), minimal bactericidal (MBC) and minimal fungicidal (MFC) concentrations using serial dilution method**.** The tested compounds were added to the nutrient medium (beef-extract broth for bacteria and wort for fungi) as solutions in dimethyl sulfoxide (DMSO) by ensuring needed concentration (0.9–500.0 μg/mL). Bacteria and fungi inoculum was inoculated into nutrient medium (the microbial loading was 10^6^ cells (spores)/1 mL). The duration of bacteria incubation was 24 h at 35 °C and the one of fungi incubation was 48–72 h at 28–30 °C. The results were estimated by the microorganism growth measured by degree of microbial turbidity in nutrient medium. Minimal inhibitory concentration (MIC) of any compound is defined as the lowest concentration which completely inhibits visible growth (turbidity on liquid nutrient medium). 

Determination of MBC and MFC. Visually transparent nutrient medium solutions were sowed on the sterile agar medium (beef-extract agar for bacteria, wort agar for fungi). The duration of bacteria incubation was 24 h at 35 °C and the one of fungi incubation was 48–72 h at 28–30 °C. In the absence of microorganism colony growth on the incubated Petri plate, minimal bactericidal (MBC) and minimal fungicidal (MFC) concentrations of the investigated compounds were identified. The test was repeated three times.

## 4. Conclusions

New 3-substituted 1-(3-hydroxyphenyl)-5-oxopyrrolidine derivatives contained hydrazone, azole, diazole, oxadiazole fragments, 2-phenoxy- and 2,3-diphenoxy-1,4-naphthoquinones were synthesized. Methyl 1-{3-[(3-chloro-1,4-dioxo-1,4-dihydro-2-naphthalenyl)oxy]phenyl}-5-oxo-3-pyrrolidinecarboxylate demonstrated potential antibacterial and antifungal activities, as determined by the diffusion and serial dilution methods. *N*'-[(4-bromophenyl)methylidene]-1-{3-[(3-chloro-1,4-dioxo-1,4-dihydro-2-naphthalenyl)oxy]phenyl}-5-oxo-3-pyrrolidinecarbohydrazide and 2-{3-[4-(1,2,3-oxadiazol-5-yl)-2-oxo-1-pyrrolidinyl]phenoxy}-3-{3-[4-(1,3,4-oxadiazol-2-yl)-2-oxo-1-pyrrolidinyl]phenoxy}naphthoquinone showed antifungal activity against *Candida tenuis* and *Aspergillus niger* at low concentrations as determined by the serial dilution method. The substitution of the methoxy fragment by *N-*containing substituents in monophenoxy substituted naphthoquinones was found to decrease their activity against *Mycobacterium luteum*. Besides, introduction of the second phenoxy substituted fragment increased the antifungal activity against *Candida tenuis* and *Aspergillus niger* at lower concentrations.
